# Spectrum of Light as a Determinant of Plant Functioning: A Historical Perspective

**DOI:** 10.3390/life10030025

**Published:** 2020-03-17

**Authors:** Oxana S. Ptushenko, Vasily V. Ptushenko, Alexei E. Solovchenko

**Affiliations:** 1 Faculty of Bioengineering and Bioinformatics, Lomonosov Moscow State University, 119234 Moscow, Russia; 2 A.N. Belozersky Institute of Physico-Chemical Biology, Lomonosov Moscow State University, 119992 Moscow, Russia; 3 N.M. Emanuel Institute of Biochemical Physics of Russian Academy of Sciences, 119334 Moscow, Russia; 4 N.N. Semenov Federal Research Center for Chemical Physics, 119991 Moscow, Russia; 5 Faculty of Biology, M.V. Lomonosov Moscow State University, 119234 Moscow, Russia; 6 Institute of Medicine and Experimental Biology, Pskov State University, 180000 Pskov, Russia

**Keywords:** regulatory and energy-supplying functions of light, action spectrum of photosynthesis, inter-leaf light intensity gradient, chloroplast photorelocation

## Abstract

The significance of the spectral composition of light for growth and other physiological functions of plants moved to the focus of “plant science” soon after the discovery of photosynthesis, if not earlier. The research in this field recently intensified due to the explosive development of computer-controlled systems for artificial illumination and documenting photosynthetic activity. The progress is also substantiated by recent insights into the molecular mechanisms of photo-regulation of assorted physiological functions in plants mediated by photoreceptors and other pigment systems. The spectral balance of solar radiation can vary significantly, affecting the functioning and development of plants. Its effects are evident on the macroscale (e.g., in individual plants growing under the forest canopy) as well as on the meso- or microscale (e.g., mutual shading of leaf cell layers and chloroplasts). The diversity of the observable effects of light spectrum variation arises through (i) the triggering of different photoreceptors, (ii) the non-uniform efficiency of spectral components in driving photosynthesis, and (iii) a variable depth of penetration of spectral components into the leaf. We depict the effects of these factors using the spectral dependence of chloroplast photorelocation movements interlinked with the changes in light penetration into (light capture by) the leaf and the photosynthetic capacity. In this review, we unfold the history of the research on the photocontrol effects and put it in the broader context of photosynthesis efficiency and photoprotection under stress caused by a high intensity of light.

## 1. The Milestones of Plant Photobiology 

The light effects on plants started to draw the attention of scientists at the end of the 18th century after Joseph Priestley’s (1772) [1] discovery of photosynthesis and Jan Ingenhousz’s (1779) [2] finding of its dependence on light (though the idea of probable light contribution to “*ennobling principles of vegetables*” was formulated half a century earlier by Stephen Hales (1727) [3]). Shortly, researchers started to wonder if the effects of light depended on its spectral composition. However, it was not clear even if the effects of light on plant (including photosynthesis itself) actually resulted from light interaction with the living plant and not from its direct action on plant constituents: Light was already known to be able to change chemicals, for example, to decompose salts, bleach oils, and reduce metal oxides. It was “*…supposed to operate directly upon the air, and to possess the power of decomposing carbonic acid, when this substance is presented to it within the pores of the vegetable tissue*” [4]. Still, alternative ideas existed, so light was believed to stimulate specifically certain “vital functions” of plants, “*enabling them to secrete from the carbonic acid presented to them the carbon required for their nutrition*” [4].

Despite its downright vitalistic nature, this standpoint (opposing the mechanistic one) was close to the current understanding and turned out to be quite productive. By the second third of the 19th century, the light dependence of carbonic acid decomposition and consequent oxygen evolution, plants greening (de-etiolation), morning leaf expansion or unfolding in certain species, irritability (e.g., of *Mimosa pudica*), transpiration, and root water uptake [4] became evident. Naturally, questions arose about the spectral component(s) of light driving those processes most actively. Thus, the violet [5], blue [6], or yellow rays [7] were assumed to be responsible for plant de-etiolation (triggering chlorophyll biosynthesis). The indigo light was supposed to give rise to phototropism [7]. The stimulating effect of light on curled cress seed germination was reasoned to decrease in a series: Blue, red, yellow, green [6]. The discrepancies between these results as well as between them and current assessments were caused by imperfect techniques both for isolating light spectral components and for documenting the light-driven physiological effects (often limited to semiquantitative assessments). Thus, colored glasses, bottles with colored solutions (e.g., those of potassium dichromate, cupric acetate, etc.), were often used as filters to isolate the needed spectral components from sunlight; optical prisms were used occasionally; and light intensity was often insufficient [8]. 

A photobiological question of special importance was that about the action spectrum of photosynthesis or, according to the terminology of the authors of 19th century, “*the power of coloured light in producing the decomposition of carbonic acid*”. Especially long-lived was the belief that “*the activity of the different rays seems to follow very closely their illuminating power*” [9], i.e., correlates closely with the spectral sensitivity of the human eye. It was based on the experiments of the American scientist John Draper (1843) [10], later reproduced by German botanist and plant physiologist Wilhelm Pfeffer (1871) [11], and became mainstream for several decades. The yellow and green light were therefore believed to be the most active. In the early 1870s, Climent Timiriazeff pointed out the mistakes in these experiments. Being inspired by fundamental ideas on thermodynamics of Mayer (1845) [12] and Tyndall (1863) [13] and empowered by spectrum analysis techniques developed by Kirchhoff and Bunsen (1860) [14] and already employed by Stokes (1864) [15,16] for solving problems of organic matter, he demonstrated that the photosynthetic action spectrum coincides with the absorption spectrum of chlorophyll. This finding implied the involvement of chlorophyll in photosynthesis. Some decades later, this point of view became generally accepted [17,18].

**Figure 1:**
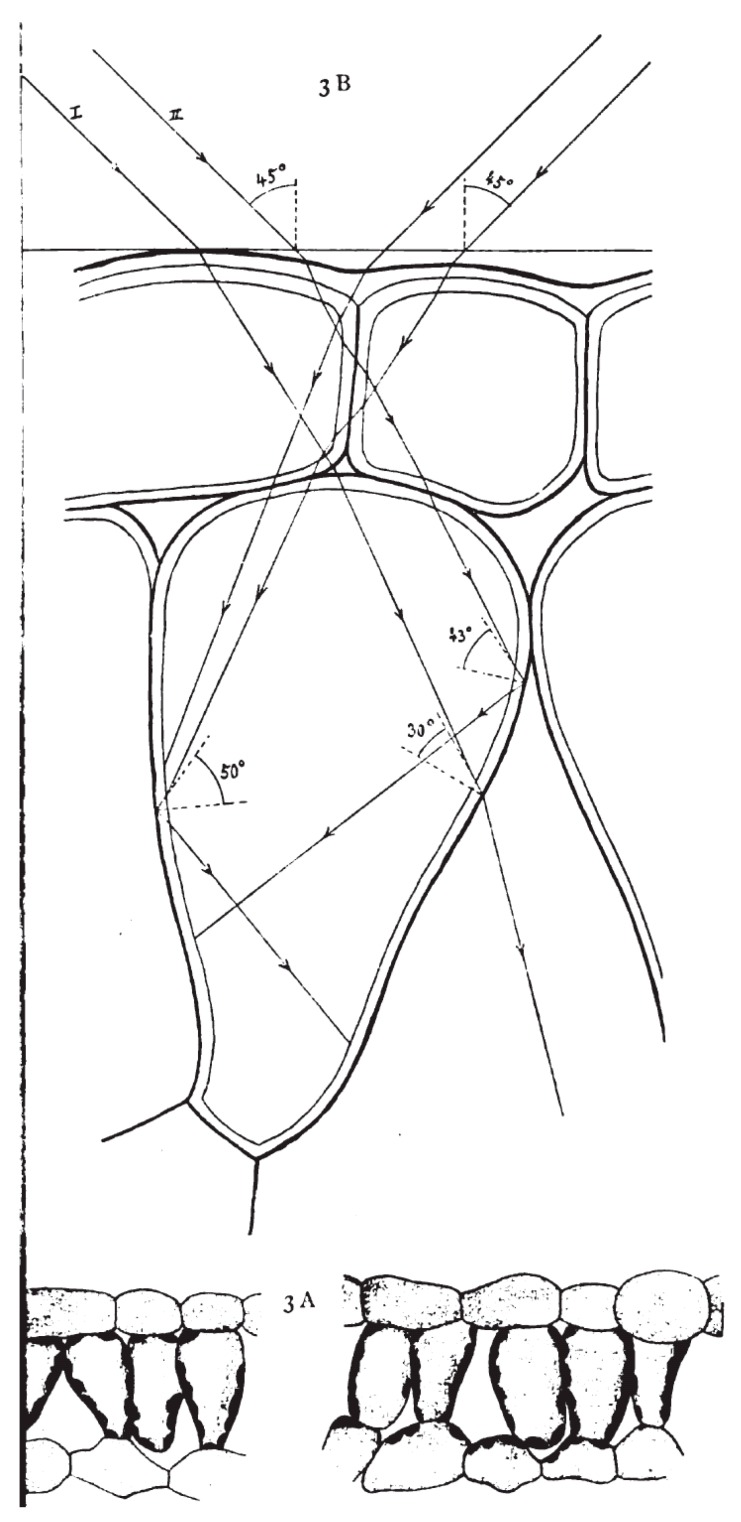
Figure from Senn’s book (1908) [22] illustrating chloroplast distribution in and light paths through *Phaseolus vulgaris* mesophyll cells.

Along with seed germination, plant de-etiolation, and carbonic acid decomposition, other issues of plant photobiology (although this field was not yet named as such) came to the scope of researchers. Here, we shall consider the example of light-dependent chloroplast movements and chloroplast redistribution patterns. The light-dependent chloroplast distribution patterns were first described by Josef Böhm (1956) [19] in higher plants (*Crassulaceae*), and the “chlorophyll grains” moving towards the light in filamentous diatoms *Oscillaria* sp. was reported by B. Frank (1871) [20]. Yet Gustav Senn is generally considered as “the pioneer of chloroplast movement research” [21] due to his seminal book “The Changes in Shape and Position of Plant Chloroplasts” [22]. In his book he summarized data on chloroplast migration in response to external stimuli, first of all to light, taking into account the optical properties of individual cells and determining light paths within the cells (Figure 1). The specialists affirm that even though the book was published more than a century ago, it incorporates a large amount of detailed knowledge regarding chloroplast movement, which is useful till nowadays [23]. After finishing his fundamental work, Senn “*retreated from the study of chloroplasts and became a researcher of the Greek philosopher, Theophrastus*” [21].

The discovery in 1959 of phytochrome photoreceptors [24] predicted earlier [25] was certainly a paramount milestone in the history of plant photobiology. This discovery allowed a line to be drawn between the regulatory and energy-supplying functions of light, both essential for photosynthesis. Below we discuss both branches of studies, i.e., presumably dealing with regulatory or “energizing” effects of the light spectrum on the photosynthetic capacity, high light stress tolerance, and productivity.

## 2. Energy-Supplying Function of Light in Photosynthesis

As it was mentioned above, Timiriazeff (1877) [17] showed that the action spectrum of photosynthesis coincided with the absorption spectrum of chlorophyll, concluding that chlorophyll is the photosensitizer for CO_2_ assimilation reaction(s); similar evidence for the participation of other pigments in photosynthesis in algae was obtained shortly [26]. Numerous photosynthetic action spectra have been obtained since then, showing more variation in algae than in higher plants (see, e.g., [27]). Still, radiation in the green range of the spectrum demonstrated the lowest ability to drive photosynthesis (at least, in green algae and higher plants). It should be stressed that the action spectra were recorded mostly with dilute algal suspensions having a low chlorophyll concentration. The data indicating the low efficiency of green light for photosynthesis also came from experiments with diluted suspensions of isolated chloroplasts (e.g., [28]). Until now, this understanding has dominated the textbooks on biology and plant physiology [29].

**Figure 2:**
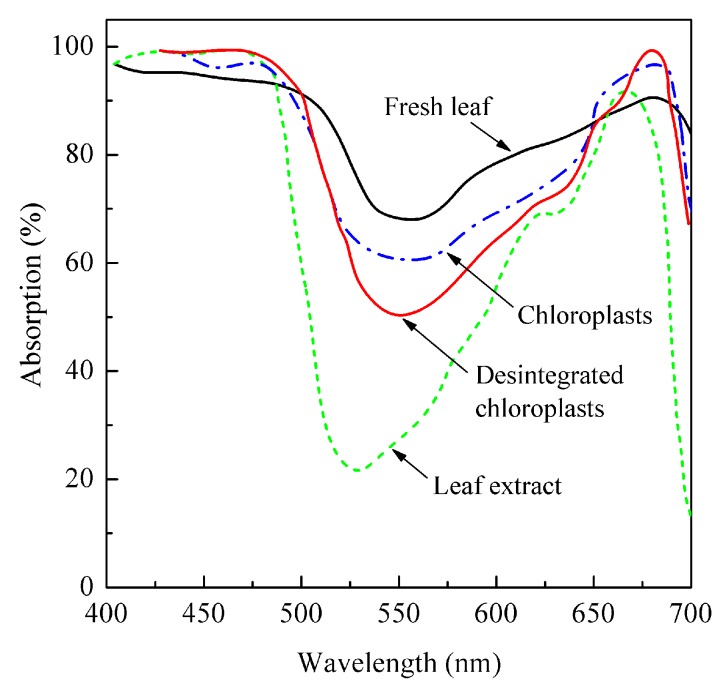
Absorption spectra of spinach leaves, isolated chloroplasts, and pigment extracts containing equivalent quantities of chlorophyll. Redrawn from [31].

However, a high (more than a half of incident light, up to 80%-90% in thick leaves) absorption of light in the green region of the spectrum by an *intact leaf* was demonstrated quite early (e.g., [30,31]; Figure 2). Notably, chloroplasts and especially pigment extracts isolated from the same leaves revealed a significantly lower green light absorption. This discrepancy eventually led to the recognition of the “usefulness” of green light for photosynthesis at the leaf level. Already, in the late 1940s to early 1950s, this idea was explicitly formulated. Thus, Strain (1950) [32] affirmed it, referring to his earlier book chapter [33] and considering light absorption in individual chloroplasts differing in size and chlorophyll content: “*Virtually all the incident red and blue light could, therefore, be absorbed by a single chloroplast containing this amount of chlorophyll, whereas most of the incident green light would be transmitted to penetrate other cellular materials… The incident green light would penetrate many chloroplasts and much cellular material before it is absorbed.*”

**Figure 3:**
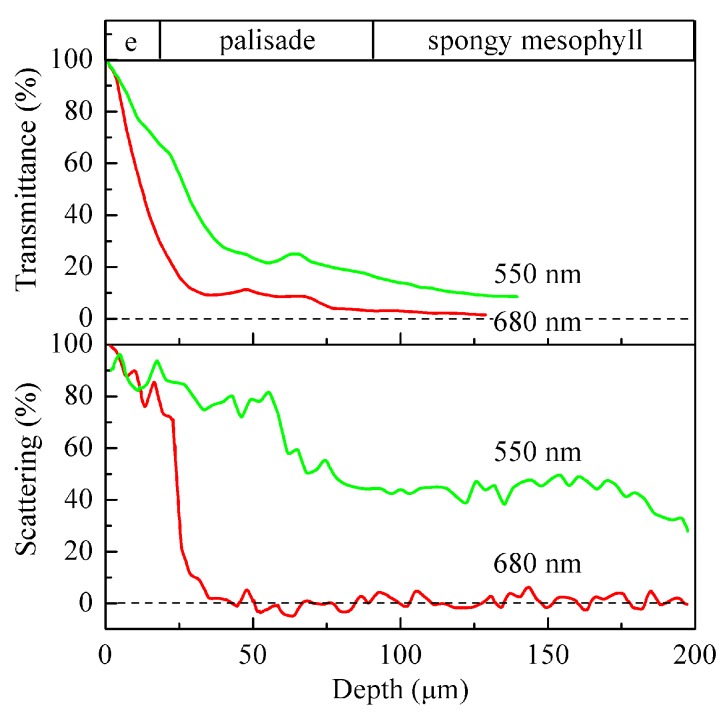
Representative distribution of transmitted (upper plot) and scattered (lower plot) light across *Medicago sativa* leaf. ‘e’ designates the epidermal layer. Redrawn from [37].

Still this idea remained a marginal concept in the field of photosynthesis for a long time. The renaissance came with new experimental capabilities for assessing the interleaf spatial distribution of CO_2_ assimilation (e.g., [34,35]) and characterization of the illumination microenvironment, both in silico [36] and by direct measurements [37,38]. Extremely elucidative data on leaf optics were obtained. To name a few, local boosting of irradiance inside the leaf due to focusing of the incident light by the epidermal cells acting as mosaic microlenses was characterized; the spectral characteristic for the directional light fluxes inside a leaf was also obtained [37]. Apart from elucidating the ‘inner world of leaf’ both literally and metaphorically, they augmented the understanding that CO_2_ assimilation does not normally follow the light gradient. Thus, most of the light was absorbed in the upper part of a leaf, i.e., within the first 20% of the leaf cross-section counting from the illuminated leaf surface (Figure 3). At the same time, the CO_2_ assimilation rate reached its maximum closer to the middle of the leaf. In contrast, the gradient of the green light irradiance evidently followed the carbon fixation rate more closely than the irradiance gradients in the red and in the blue. Actually, strong absorption of red and blue light in the uppermost layers of a leaf would shade the lower cell layers nearly completely. As a result, they would not contribute to photosynthesis if it was not for the green component in the daylight spectrum.

The long-noted high absorption of dense leaves in the green, making them look rather greenish black than green, became fully substantiated and reasonable from the standpoint of biology. So, the feeble idea from the early 1950s that the incident green light penetrates to the deeper layers of a leaf where it drives photosynthesis gained its complete quantitative form and is beyond doubt nowadays [29,39], though it does not seem to be widely known outside a narrow circle of specialists.

Less unambiguous is the probable energy-supplying function of far-red (FR) light. It is even less absorbable by a leaf than green light. The low absorbance of leaf for FR light is employed by the “shade avoidance” phytochrome-based signaling system, but could it serve as an additional energy source for leaves shaded by the canopy or for leaf cells overlaid by the upper mesophyll layers? It is difficult to conceive since photosystem I (PSI) is scarcely able to catch light beyond 710–720 nm and photosystem II (PSII) is even less able. Some organisms overcame this by including into the pigment apparatus exotic long-wave chlorophylls, e.g., chlorophyll *f* in cyanobacteria [40]. However, it would be much more elucidating to find out if the absorbed FR light gets utilized for photosynthesis in “normal” (lacking the exotic chlorophylls) higher plants. Although long wavelength chlorophylls located in the photosystem I core and light-harvesting complex were reported [41], there was no firm evidence for FR light contribution to photosynthesis. Some support for this appeared recently from the studies on the conventional model plants *Arabidopsis thaliana*, *Nicotiana tabacum*, and *Alocasia odora* [42]. The FR light (740 nm) added to fluctuating red light (630 nm) was shown to increase the quantum yield (Φ_PSII_) of photochemical reactions in PSII and CO_2_ assimilation (*A*) due to additional PSI activation. It was found to facilitate the electron transfer reactions on the PSI acceptor side and hence to increase the thylakoid membrane conductivity for protons mediated by ATP-synthase activation. All this allowed for faster relaxation of nonphotochemical quenching after an abrupt decrease in red light intensity and more efficient use of the weak red light. So, the mechanism of boosting photosynthesis with FR light, at least on the background of fluctuating photosynthetically active radiation fluxes, has been demonstrated. One may suppose that this FR light-dependent boost evolved as an adaptation to the peculiar illumination environment in the canopy. The gain might be sizeable since the simulations predict 13% to 32% (depending on the temperature and chilling tolerance) of daily canopy-level carbon fixation losses due to a lagging photoprotection disengagement [43].

One can raise the question of whether this effect of the FR light is more “regulatory” or “energy-supplying”. Actually, the FR light effect on CO_2_ assimilation is achieved by increasing the efficiency of red light utilization (i.e., what one could refer to as an FR light-dependent regulation of electron transfer reactions) rather than by utilizing FR light energy per se. It is difficult to distinguish between these modes of action since photosynthetic processes are fine-tuned by changing light conditions. The problem is that the fine-tuning employs the same molecular structures that are being regulated. Therefore, it seems reasonable to attribute the FR light-induced photosynthetic activity to the “energy-supplying” mode (elaborated on in this section) and to proceed to the next section on the canonic photoregulatory systems.

## 3. Spectrum-Dependent Light Regulation of Photosynthesis

In the previous section, we pointed out that the role of certain spectral components changes while the beam penetrates deeper into the leaf. In other words, this role is determined by the transverse light gradients, which are in turn photoregulated. Among the mechanisms of acclimation to the fluctuating light environment, higher plants (and many lower plants) possess light-dependent chloroplast movement, or chloroplast photorelocation. In the introduction, we briefly touched on this issue, starting from the findings by Böhm (1856) [19] and Frank (1871) [20] and continued by Senn (1908) [22]. Below, two interrelated stories of the spectral dependency of light acclimation and its physiological effects will be told.

### 3.1. Spectral Dependency of and Photoreceptors for Chloroplast Photorelocation Movement 

Before the beginning of the “photoreceptor era”, researchers did not see the need for any additional light-dependent regulatory systems for chloroplasts: “*…long ago it was believed self-evident that chloroplasts orient to light by finding out where the best or least illuminated places of the cell are. In order to discover this, they must absorb the light and ‘measure’ it*” [44]. The early data on the action spectra of chloroplast photorelocation seemed to support this belief. The blue-violet spectral range, namely, 450–480 nm, was shown to be the most active followed by a smaller peak in ultraviolet-A UV-A at about 360 nm [45,46,47,48]. Certain organisms, e.g., *Vallisneria* [49], revealed a very low sensitivity to red light or displayed a distinctive red/FR light reversible (i.e., phytochrome-dependent) response (*Mougeotia*, [50]). Some discrepancies between the features of the obtained action spectra allowed the corresponding effects to be ascribed to carotenoid chromophores (e.g., [45]), which was in line with the postulated autoregulation of the chloroplast relocation.

By the early 1960s, the evidence accumulated suggested that the photoreceptors that determine chloroplast movement, putatively flavins [51], localize in the cytoplasm but not in the chloroplasts [50]. However, exceptions were also known. Thus, the engagement of phytochromes containing bilin chromophore in chloroplast-positive phototaxis was previously shown for the green alga *Mougeotia*, a classic model plant for chloroplast photorelocation studies [50]. The idea of riboflavin as a photoreceptor came into chloroplast photorelocation concepts from studies in a closely related field—phototropism. Riboflavin was suggested to serve as a photoreceptor mediating the classic example of the phototropic movements—the curving of *Avena* coleoptiles—instead of the previously assumed carotenoids in 1949 [52]. The belief that the hypothetic flavin photoreceptor is pivotal for the chloroplast photorelocation response strengthened during the next decades. Nevertheless, in the 1970s, the possibility could not be “*definitely excluded… that the blue-light absorbing photoreceptor pigment is a carotenoid rather than a flavin*” [53], and even in the 1980s, a careful wording like “*All the facts presented are not in contradiction with the assumption that flavin pigment acts as a photoreceptor controlling the position of chloroplasts*” was still needed [54].

Today, we know that riboflavin is the chromophore of photoreceptor phototropin [55], mediating both positive and negative blue/UV-A light-dependent chloroplast phototaxis (referred to as accumulation and avoidance photorelocation, respectively) in most plant species. The recent study of Hermanowicz et al. (2019) [56] surprisingly revealed UV-B light-dependent chloroplast accumulation and avoidance photorelocation in *Arabidopsis* leaves. Moreover, the canonical UV-B photoreceptor UVR8 is not involved in controlling UV-B induced photorelocation; rather, it is also mediated by phototropins. In cryptogam plants (e.g., green algae *Mougeotia scalaris* and *M. caldariorum*, moss *Physcomitrella patens*, or fern *Adiantum capillus-veneris*), red light-induced phytochrome- or neochrome-mediated chloroplast movement was found [57].

### 3.2. Chloroplast Photorelocation Movement in Acclimation to Environmental Stimuli 

Though the effect of chloroplast photorelocation movement on light energy capture has been experimentally studied soon after its discovery [58,59], the first quantitative investigation of its effect on the carbon assimilation rate was probably performed in the 1950s [60]. However, this effect appeared to be restricted to low irradiances. The lack of influence of the chloroplast arrangement on photosynthesis led Jan Zurzycki to the hypothesis that a decrease of light absorption at a high ambient irradiance should limit its harmful effect. Omitting the history of this idea’s development, we only point to the work of Kasahara et al. (2002) [61], offering direct evidence from *A. thaliana* mutants defective in chloroplast avoidance movement: An impaired avoidance response made plants more susceptible to photodamage. Although there are recent data questioning the efficiency (at least in high irradiance-grown plants [62,63]) or the need of the avoidance response and even assuming the advantages of its lack [64], its significance for protecting the photosynthetic apparatus from very intense light remains evident [65].

The crucial role of chloroplast avoidance movement in plant photoprotection was proposed for certain plant species revealing the highest photoinduced changes in the optical properties of their leaves [66,67]. This protective mechanism of chloroplast avoidance movement is partly caused by a decrease in the overall light absorption by a leaf, but this decrease is quite low. Thus, Davis et al. (2011) [68] working on 24 plant species showed that the changes in red light transmittance comprised ca. 2% to 6% on average for sun- and shade-grown leaves, respectively. Eleven species studied by Königer and Bollinger (2012) [69] revealed 1% to 12% or 7% to 20% changes in response to the accumulation- or avoidance-type movement, respectively. The only exception was constituted by *A. thaliana* (23% and 38%), which seem to “exploit” the chloroplast photorelocation most efficiently. The only species comparable with and even surpassing *A. thaliana* are *Tradescantia* species, which reveal two- (*T. albiflora*, [66]; *T. sillamontana*, [67]) to four-fold changes (shade-grown *T. fluminensis* leaves, [67]).

However, much more essential for the photoprotective effect is the change in the inter-leaf light intensity gradient(s) and hence in the light absorption transverse profile. The net result of this is a redistribution of the “excitation pressure” on the photosynthetic apparatus, altering the risk of photoinhibition at a different depth within a leaf. Thus, a quite small change in the overall light absorption of 8.4% should lead to a decrease (up to 1.6-fold) in light absorption in the upper one third of the leaf cross-section and a coinciding increase (up to 1.7-fold) of light available to the cells in the lower two thirds [70]. As a result, the photosynthetic apparatus of the upper mesophyll cells can avoid photoinhibition while the deep cells get a chance to capture more light and increase their photosynthetic performance.

**Figure 4:**
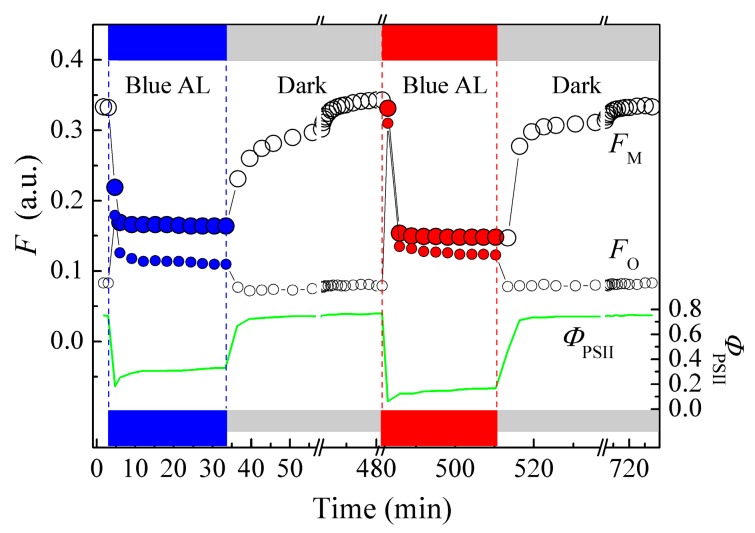
The time course of saturating pulse-activated (*F*_M_) and transient chlorophyll fluorescence (*F*_O_) at blue or red actinic light of moderate irradiance (155 μmol photons m^−2^s^−1^) and photosystem II efficiency (Φ_PSII_) in *T. fluminensis* leaves [71].

The spectral dependence of the chloroplast photorelocation may lead to another effect, probably less significant for wild plants but potentially essential for crop plants grown under artificial illumination. Namely, the chloroplast photorelocation movement is not activated in higher phototropin-containing plants illuminated by light containing a decreased proportion of energy in the blue spectral range. This makes plants prone to photodamage and decreases the plant’s photosynthetic capacity, if other protective mechanisms could not fully compensate the impaired photorelocation. Thus, irradiating *T. fluminensis* leaves by “pure” red light of moderate irradiance (100–500 μmol photons m^−2^s^−1^) decreased Φ_PSII_ 2–2.5-fold compared with the leaves irradiated by blue light of the same intensity (Figure 4; [71,72]). However differences in the photoinhibitory effects of blue and red light remained negligible (if any) up to relatively high irradiance, at least in *T. fluminensis* (up to 500 μmol photons m^−2^s^−1^, [71]) and *A. thaliana* leaves (up to 1350 μmol photons m^−2^s^−1^, [62]). Moreover, some studies indicate even higher photoinhibition by blue than by red light in the same species at comparable irradiances ([72,73]).

It is important to emphasize that not only the light gradient across the leaf and per-chloroplast irradiation are influenced by chloroplast photorelocation but also all the intercellular arrangement of organelles. Thus, mitochondria also exhibit a blue-light-dependent redistribution following the chloroplasts. Obviously, this response is controlled by phototropins and likely other signals deriving from photosynthesis but follows a distinct timing [74].

## 4. The Spectral Effects on a Whole-Plant Scale 

Although the experiments on growing plants under artificial light (provided by petrol lamps) were started already by Famintzin (1865) [75], the integral effects of the irradiation spectrum on plant growth drew tremendous attention in recent decades due to the booming development of artificial lighting and glasshouse systems. Since modeling the sunlight (“natural”) spectrum has been, for a long time, a difficult problem (which has not been completely solved till now), various approaches to obtaining “white light” have been used both in scientific research and the industrial cultivation of plants. The studies have been a hotspot since the early 1990s, when light-emitting diodes (LEDs) and the first LED lamps became widely accessible [76,77,78,79]. Initially, only red LEDs were available; however, their narrowband (full width at half maximum, FWHM, of 20–25 nm) spectrum overlapping with the longwave photosynthetic action spectrum maximum was considered as an advantage due to its expected high efficiency [80]. Nevertheless, the first experiments showed that narrowband red (peaking at 630–670 nm) irradiation was insufficient for healthy plant growth and development. Thin elongated stems and petioles [81,82,83], retarded, reduced or even inhibited flowering [84,85], as well as impaired photosynthesis [86] pestered the growing systems employing red LED light.

These results stimulated both the use of light sources emitting a wider number of spectral components and study of their physiological role. Adding blue light eliminated most of the higher plant growth deficiencies caused by substituting white light by narrow-band red irradiation, though there were large variations between different species (Appendix A, Table A1). In algae, the light spectrum also affected biomass accumulation and the chemical composition [87,88,89]. It became clear later that “red plus blue” still could not completely substitute white light, so other spectral components and the physiological effects mediated by them came into the focus (for a review, see Appendix A, Table A1).

Most of the effects appeared to be photoreceptor dependent. The LED era in plant biology coincided with the key discoveries of photoreceptors. Although the physiological effects of blue light had been known for at least half a century, the first blue light photoreceptor cryptochrome, regulating hypocotyl growth, circadian rhythms, and flowering in *A. thaliana*, was discovered only in 1993 [90]. In the next few years, the rhodopsin-like photoreceptors were found [91], and a set of flavin-dependent receptors, starting from phototropin [55]. The latter appeared to control chloroplast photorelocation movement [92,93], as well as stoma opening, nucleus transposition, leaf alignment, position of leaves, fast inhibition of hypocotyl growth [94], and the expression of stress-related photoprotective proteins [95]. The phototropin discovery was followed by the finding of a series of related blue light receptors, such as chimeric phytochrome and phototropin-related neochrome [96], BLUF (blue-light using FAD) sensors [97], and aureochromes [98]. All these blue light photoreceptors are also sensitive to UV-A light [94]. The molecular nature of UV-B sensors remained unknown for a long time, but recently, the UVR8 protein (originally identified as a regulatory protein for ultraviolet-B-triggered signal transduction) was shown to provide UV-B photoreception, with two tryptophan residues serving as the chromophores [99].

The green light signal reception and transduction mechanisms remain much more enigmatic, though its physiological effects are quite evident now. They involve facilitating or canceling triggering by other spectral components possessing their own photoreceptors, affecting seed germination, plant morphogenesis, shade avoidance, cell division and elongation, dry mass accumulation, transpiration, and regulation of the endogenous level of phytohormones [100]. Probably, green light employs red and/or blue light receipting and transducing systems, which chromophores absorb to some extent or could be transformed to green light-absorbing forms. Thus, the involvement of cryptochrome in green light perception was first shown in 1995 [101] and repeatedly confirmed later for blue/green light ratio sensing as well [102].

Along with the regulatory effects of the spectral components other than the red and blue, their energy-supplying effect manifests itself in plants grown under the narrowband light. The role of green light in supplying deep mesophyll layers and the inner canopy leaves with energy for photosynthesis was mentioned above. Notably, it became evident only in the last three decades and its significance is often underestimated till now. It was experimentally demonstrated that in the plants grown under the “red plus blue” light forming a dense canopy with mutual shading of leaves, this could lead to a loss of up to 40%–50% of the growth efficiency due to deterioration of the radiation distribution within the plant canopy [103].

## 5. The Promise for Improving the Spectral Response of Photosynthesis in Plants

The role of the spectral components of sunlight in energy supply and regulation of plant physiological functions has become increasingly distinct over recent years. Thus, many features of the photosynthetic machinery perceived as hallmarks of their imperfection turned out to be important regulatory and/or acclimatory traits. However, the bioengineering-based approaches for improvement of the light use efficiency of plants were developed recently. Among the most radical are the proposals to expand the spectral range used in photosynthesis into the near-infrared range (up to 1100 nm) by engineering bacteriochlorophylls into plant light-harvesting antenna [104,105].

Currently implemented approaches to increase the solar energy-to-biomass conversion efficiency comprise improving the light distribution within the plant canopy and/or leaf mesophyll tissue by tuning the chlorophyll content. Plant mutants with ca. two-fold reduced chlorophyll content revealed a higher net photosynthesis and photosynthetic N use efficiency [106] as well as up to 25% higher biomass accumulation [107]. This effect was presumably achieved due to (i) the prevention of shading of deeper leaf layers and (ii) alleviation of excess absorption of sunlight and hence wasteful non-photochemical dissipation of the absorbed energy. Although the experiments showed the advantages of a lower chlorophyll content, the question remains why, in nature, plants still accumulate apparently excessive amounts of chlorophyll. It seems especially enigmatic given that variation of the chlorophyll content is the most flexible and widespread mechanism of plant acclimation. One can assume that the Achilles’ heel of plants with artificially lowered chlorophyll content will be revealed further in more detailed studies.

Less radical methods of engineering light capture in plants include modulation of the operation of photoreceptors or other regulatory systems. Thus, chloroplasts in phototropin-deficient mutants could be anchored at a certain arrangement (corresponding to, e.g., the accumulation response, avoidance response, or dark-adapted state), optimizing light absorbance by and its intensity gradient within the leaf. In some cases, this can lead to enhanced photosynthesis and plant biomass production [64]. An even more elegant approach is engineering the phototropin photocycle for the photoreceptor performance. In phototropins, light induces the formation of a covalent adduct between the chromophore and the protein, which decays thermally in darkness. Site-directed mutagenesis allows an acceleration or slowing down of this decay, which in turn affects the rate and extent of chloroplast accumulation and their avoidance movement, as well as leaf expansion and positioning efficiency, increasing biomass production [108]. A similar effect may be achieved by modulating the rate of light-dependent changes in non-photochemical dissipation of the absorbed energy rather than in light absorbance itself [109].

Nevertheless, the most radical approaches to improving absorbance of or light use efficiency by a leaf proposed till now rather choose the way of simplifying the photosynthetic system at the cost of reducing its flexibility. Future trials will show the actual efficiency of this approach.

## 6. Conclusion 

Plant life fully depends on the availability of light. Integral to it is the multifaceted regulatory role of light, so the light spectrum serves as a multimodal regulatory signal. However, the meaning of distinct spectral components for many different aspects of plant development and physiology remains unclear; some breakthroughs in the area came only recently (e.g., FR light usage in photosynthesis in higher plants, see [42]). Of special interest is the aspect relating the spectral effects with plant evolution. Thus, a highly debated question of the evolutionary theory, “*to what extents modern organisms are suboptimal*” [110], has applications in plant physiology. Thus, we still do not understand entirely why plants “prefer” using a few spectral components over others, and is this preference related to some physical limitations of the photosynthetic apparatus or it is just an ‘evolutionary accident’ (for a review, see, e.g., [111])? A vivid example of a hot research topic in this field is the significance of green light for photosynthesis, long disregarded and hence underexplored. Recent insights into the control of assorted aspects of plant life by spectral components of incident radiation contribute not only to the basic knowledge of plant physiology and evolution. They are also of direct relevance for the efficient growing of crop plants under artificial illumination, which becomes increasingly important for the sustainable development of civilization.

## Appendix A

**Table A1:** Dry mass (DM) or fresh mass (FM) accumulation, seeds mass (SM), soluble sugars (SS), soluble proteins (SP), and rates of photosynthesis (RP) of red-plus-blue light (RBL)-grown plants compared with pure red (RL)-, blue (BL)-, or polychromatic broadband light (PCBL)-grown plants. A: Species; B: BL portion in irradiance, BL/(RL+BL) (the range within which the growth rate increases with BL portion in irradiation); C: Total growth irradiance (μmol photons∙m^−2^∙s^−1^); D: Photoperiod (h); E: Age of analyzed plants (days); F: Analyzed parameter; G, H, I: The relative change of the parameter compared with pure RL (G), pure BL (H), or PCBL (I); J: The source of PCBL: cool-white fluorescent lamps (CWF), dysprosium lamps (DyL), daylight fluorescent lamps (DWF), fluorescent lamps (FL; no information about FL model or light spectrum is provided in the paper), cool-white fluorescent plus incandescent lamps (CWF + Inc), metal halide lamps (MH), high pressure sodium lamps (HPSL); K: References.

	A	B	C	D	E	F	G	H	I	J	K
1	*Cucumis sativus* L.	7–50%	100	16	17–22	RP	3	1.4			[86]
2	*Raphanus sativus* L.	5–10%	200	16	25, 50	RP DM	1.8–2.2 1.6				[83]
3	*Raphanus sativus* L., *Lactuca sativa* L., *Spinacea oleracea* L.	50%	300	18, 18, 12 ^1)^	21	RP DM	2.4, 0.93, 0.94 ^1)^ 1.6–2.4, up to 8 ^3)^		0.97, 1.11, 0.82 ^1)^ 0.5–0.95	CWF	[112]
4	*Raphanus sativus* L., *Lactuca sativa* L., *Capsicum annuum* L.	0–12%	200	16	21, 21, 21 ^3)^	DM	1.1–2.0	1.15–1.75			[81]
5	—“—	–“–	500	16	–“–	DM	1.2–1.65	1.15–1.8			—“—
6	*Brassica campestris* L.	14%	150	12	28 ^3)^	FM DM SS SP	1.8 1.15 0.71 1.15	1.8 1.19 0.51 1.08	2.0 1.23 1.32 1.05	DyL	[113]
7	*Triticum aestivum* L.	10%	350	24	15–70	RP DM SM	2.1–2.3 1.4–1.7 1.9		0.7–0.1 (0.55–0.67) ^4)^ 0.6–1.0 (0.4–0.9) ^4)^ 0.8 (0.7) ^4)^	CWF	[84]
8	*Doritaenopsis* hort., in vitro	50%	70	16	14–56	FM, DM	1.3–1.7	1.3–2			[114]
9	*Withania somnifera* (L) Dunal., in vitro	50%	30	16	35 ^3)^	FM, DM	2.6	2.8			[115]
10	*Lactuca sativa* L.	0–59%	170	12	28 ^3)^	FM DM	0.25 0.3				[116]
11	*Brassica campestris* L.	11%	80	12	60	FM DM	0.9 0.7	0.8 1.1	3.8 2.3	FL	[117]
12	*Lactuca sativa* L.	10%	325	16	21	FM DM			1.17 1.14	CWF + Inc	[77]
13	*Capsicum annuum* L.	1%	300	12	21 ^3)^	DM	1.0–1.7		0.75–0.9	MH	[118]
14	*Brassica chinensis* L.	14%	100	24	15, 27	DM SS			0.5–1.08 0.2	HPSL	[119]
15	—“—	–“–	400	–“–	–“–	DM SS			0.76–0.86 0.4–0.6	HPSL	—“—

^1)^ the set of values correspond to species listed in the 1^st^ column ^2)^ the value corresponds to storage roots of *Raphanus sativus* ^3)^ duration of RL, RBL, or BL light treatment ^4)^ the values correspond to 1% BL.
